# An effect size for comparing the strength of morphological integration across studies

**DOI:** 10.1111/evo.14595

**Published:** 2022-08-22

**Authors:** Mark A. Conaway, Dean C. Adams

**Affiliations:** ^1^ Department of Ecology, Evolution, and Organismal Biology Iowa State University Ames Iowa USA

**Keywords:** Integration, morphometrics, macroevolution, phenotypic evolution

## Abstract

Understanding how and why phenotypic traits covary is a major interest in evolutionary biology. Biologists have long sought to characterize the extent of morphological integration in organisms, but comparing levels of integration for a set of traits across taxa has been hampered by the lack of a reliable summary measure and testing procedure. Here, we propose a standardized effect size for this purpose, calculated from the relative eigenvalue variance, $V_{rel}$. First, we evaluate several eigenvalue dispersion indices under various conditions, and show that only $V_{rel}$ remains stable across samples size and the number of variables. We then demonstrate that $V_{rel}$ accurately characterizes input patterns of covariation, so long as redundant dimensions are excluded from the calculations. However, we also show that the variance of the sampling distribution of $V_{rel}$ depends on input levels of trait covariation, making $V_{rel}$ unsuitable for direct comparisons. As a solution, we propose transforming $V_{rel}$ to a standardized effect size (*Z*‐score) for representing the magnitude of integration for a set of traits. We also propose a two‐sample test for comparing the strength of integration between taxa, and show that this test displays appropriate statistical properties. We provide software for implementing the procedure, and an empirical example illustrates its use.

Organismal phenotypes are the outcomes of complex developmental and evolutionary processes, where selective forces influence multiple traits simultaneously. When biological factors such as genic, ontogenetic, developmental, or functional linkages elicit concomitant changes in more than one trait, phenotypic correlations will ultimately arise. “Morphological integration” (Cheverud, 1982; Klingenberg, 2008; Olson & Miller, 1958; Wagner, 1984) describes the tendency of traits to covary with one another, either across an entire organism or within particular anatomical structures (Klingenberg, 2014). Morphological integration can have a profound influence on phenotypic evolution, as it constrains phenotypic variation in certain directions, and thus affects the extent to which organisms may respond to selection (Goswami et al., 2014, 2016; Pavlicev et al., 2009; Wagner, 1984; Wagner & Altenberg, 1996; Zelditch & Goswami, 2021). As such, characterizing patterns of morphological integration, and comparing these patterns across taxa, is paramount for our understanding of how selection shapes patterns of phenotypic diversification across taxa and lineages (Klingenberg, 2014).

In some instances, patterns of trait covariation are unevenly dispersed across traits, resulting in integration that is concentrated within subsets of traits that are less correlated with other subsets of traits. In these cases, modularity is displayed (Adams, 2016; Cheverud, 1982; Hallgrimsson et al., 2009; Klingenberg, 2008; Klingenberg & Marugán‐Lobón, 2013; Wagner & Altenberg, 1996; Zelditch & Goswami, 2021). Both integration and modularity affect the degree to which phenotypic variation is exposed to selection, thereby altering phenotypic evolvability (Hansen & Houle, 2008; Wagner & Altenberg, 1996). Thus, the integration within and among sets of traits influences the way in which phenotypic variation arises and how diversification proceeds. Integration may also be observed at multiple levels of organization, where the integration of traits at one level is influenced by the association of patterns and selective processes at lower levels (Hallgrimsson et al., 2009; Klingenberg, 2014). Based on this observation, it has been suggested that integration should be considered as both a generative force as well as a resultant pattern: the former describing the propensity to influence covariation at higher levels, and the latter being the observable pattern of integration among traits linked by a common function or developmental pathway (Hallgrimsson et al., 2009) see also Klingenberg (2014).

Because morphological integration arises from the interaction among parts, deciphering how much traits covary (i.e., What is the magnitude of integration?), describing the manner in which those traits are integrated (i.e., What is the pattern of trait covariation?), and identifying the causes of trait covariation (i.e., What mechanisms generate integration?), are questions that have garnered considerable attention in evolutionary biology. Olson and Miller's pioneering (1958) work espoused a research paradigm to interrogate these questions, which contemporary quantitative morphologists have adapted and utilized in subsequent investigations. However, as with all studies in quantitative morphology, such inquiries should not be performed on a haphazard collection of measurements, as little biological insight may be gleaned from summarizing a set of arbitrary traits with no known connection with one another. Rather, an understanding of the patterns of phenotypic integration, and their underlying causes, can only be attained through evaluating traits selected on biological grounds based on their biomechanical, functional, or developmental properties. In the work that follows, we advocate this philosophy, and assume that the researcher has first scrutinized their choices of phenotypic attributes prior to any evaluation of integration between them.

With respect to quantification, various analytical approaches have been proposed for characterizing patterns of integration and modularity in multivariate phenotypes (e.g., Magwene 2001; Bookstein et al. 2003; Klingenberg 2009; Adams 2016 reviewed in Goswami & Polly 2010; Haber 2011; Zelditch & Goswami 2021). Most summarize the information contained in the p×p trait covariance matrix in some manner (but see Bookstein 2015). For instance, the degree of integration in a set of phenotypic traits has been estimated using the mean absolute pairwise correlation, the mean squared pairwise correlation (e.g., Cheverud et al. 1989; Cane 1993), or summary values based on the eigenvalues of the trait covariance (or correlation) matrix (Hansen & Houle, 2008; Haber, 2011; O'Keefe et al., 2022; Pavlicev et al., 2009; Shirai & Marroig, 2010; Van Valen, 1974; Wagner, 1984). Many studies have successfully deployed these approaches to characterize the extent to which morphological integration is displayed across traits in particular taxa (e.g., Grabowski & Porto 2017; Conaway et al. 2018; Machado et al. 2019; Jung et al. 2020b).

The present paper is concerned with a related question: For a set of phenotypic traits that share common selective pressures, does the strength of integration in one species differ from that displayed in another? Biologically, there are several reasons why differences in the strength of integration may be exhibited. For instance, changes in the magnitude or direction of selective pressures may result in alterations in the degree to which traits covary, and are thus integrated (see Hanot et al. 2018). Likewise, traits specialized for a single biomechanical task in one species may display greater integration than the same traits in taxa whose functional repertoire is more diverse (Hanot et al., 2017). Figure 1 provides several hypothetical examples for how such patterns manifest visually. First, consider the scenario where two traits interact biomechanically, and contribute to a common function (Fig. 1a). In this case, the two traits exhibit relatively strong covariation, resulting in a high pairwise correlation, and a tight 95% confidence ellipse. By contrast, Figure 1B shows a scenario where two traits are functionally independent. Here the two traits display low levels of covariation, and their 95% confidence ellipse is more circular.

**Figure 1:**
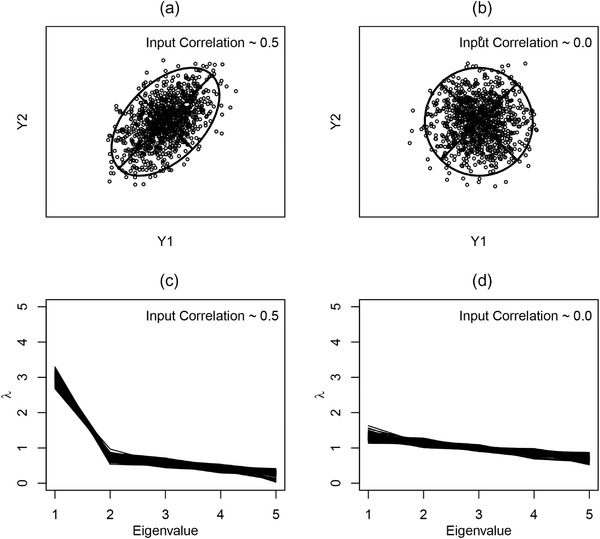
(a) Bivariate association between two hypothetical traits (ρ=0.5) with 95% confidence ellipse and major and minor axes displayed. (b) Bivariate association between two hypothetical traits (ρ=0.0) with 95% confidence ellipse and major and minor axes displayed. (c) Dispersion of eigenvalues of 100 trait covariance matrices for 5 traits each, generated with an input correlation between traits of ρ=0.5. (d) Dispersion of eigenvalues of 100 trait covariance matrices for five traits each, generated with an input correlation between traits of ρ=0.0.

Mathematically, these concepts extend to higher dimensions. When a phenotypic dataset is more highly correlated, it will form an ellipsoid in the multi‐dimensional trait space; whereas the pattern will be more spherical for a set of traits that are more independent of one another. The degree to which the scatter of multivariate data is circular or elliptical may be described by a test of sphericity (John, 1972), or by the relative lengths of the axes of the multi‐dimensional confidence ellipse circumscribing the data. The latter are found as the eigenvalues of the trait covariance matrix. For a set of highly correlated traits, the first eigenvalue will dominate, whereas for independent traits the eigenvalues will be more similar to one another. This property may be seen in Figure 1C and D, where a plot of the eigenvalues for five highly correlated and five independent traits are displayed respectively. In the case of the former, traits that covary highly exhibit a large dominant eigenvalue, followed by many smaller eigenvalues (Fig. 1c). By contrast, independent traits display a set of eigenvalues that are more evenly distributed, indicating lower overall covariation between traits. Indeed, the hypothetical examples in Figure 1 underscore the well‐known notion that highly correlated—and thus integrated—traits, will lead to a concentration of phenotypic variation in a few dominant dimensions, whereas less integrated traits will result in phenotypic variation that is more evenly dispersed across more dimensions of the dataspace (Pavlicev et al., 2009; Wagner, 1984). From this it follows that differences in the values of the eigenvalues (or more precisely, the variance or dispersion among eigenvalues) can provide the basis for summary measures of the strength of integration in phenotypic datasets.

Because of this property, several eigenvalue dispersion indices have been proposed as metrics of integration (Haber, 2011; O'Keefe et al., 2022; Pavlicev et al., 2009; Shirai & Marroig, 2010; Van Valen, 1974; Wagner, 1984), with each summarizing the variation across eigenvalues in slightly different ways. An obvious question then, is how do these different measures perform relative to one another? Simulations have shown that some indices associate highly with the mean absolute correlation across traits, implying that those indices may adequately summarize levels of morphological integration (e.g., Pavlicev et al. 2009; Haber 2011). Other studies have evaluated the effect of sample size on eigenvalue dispersion indices, demonstrating that estimates of integration tend to be more stable at larger sample sizes (Grabowski & Porto, 2017; Jung et al., 2020a; Watanabe, 2022). One recent study (Watanabe, 2022) evaluated several properties of the relative eigenvalue variance: $V_{rel}$, a metric that ranges from 0 → 1 (and is mathematically related to the test of sphericity: see Watanabe 2022). Here, it was observed that for independent traits (i.e., no integration), $V_{rel}$ remained stable, and close to zero, over a wide range of both sample size (*N*) and variable number (*p*). Indeed, this is an important property for integration indices, as it suggests that estimates of integration are not conflated with other attributes of the data that do not characterize the underlying covariance structure of the variables (see Adams 2016; Adams & Collyer 2016, 2019a). However, to date, a comprehensive evaluation has not been conducted across the set of eigenvalue dispersion indices. Thus, it remains unclear whether any of these indices are, in fact, appropriate for characterizing levels of integration in phenotypic datasets, and which may be used as the basis of comparison of the strength of integration across datasets.

In this article, we conduct such a comparison, and find that for independent variables, only the relative eigenvalue variance, $V_{rel}$, is capable of accurately characterizing integration patterns stably across different sample sizes and numbers of variables. We also confirm earlier findings of Watanabe (2022), and show that across a range of input levels of trait covariation, estimates of $V_{rel}$ reliably represent those values as well. However, we then examine the sampling distribution of $V_{rel}$, and find that the variance of this measure, and the skewness of its distribution, depend on the input level of trait covariation. Specifically, the variance of $V_{rel}$ is not constant, but decreases as $V_{rel}$ tends toward its extreme values. This observation compromises direct statistical comparisons of $V_{rel}$ for evaluating the strength of integration across datasets. As a solution to this problem, we propose use of a standardized effect size for $V_{rel}$, based on Fisher's *Z*‐transformation (Fisher, 1915, 1921). This effect size alleviates the above‐mentioned shortcomings, and results in a more stable sampling distribution of the statistic. The result is that this measure may be treated as a measure of the strength of integration in phenotypic datasets, which may thus be used for comparing levels of integration between species. To this end, we provide a two‐sample testing procedure for comparing the strength of integration between datasets. Simulations demonstrate that our procedure displays desirable statistical properties (i.e., appropriate type I error, low model misspecification, and high power). Software for implementing the approach may be found in the R‐package geomorph (Adams et al., 2022; Baken et al., 2021).

## Methods and Results

### COMPARISON OF EIGENVALUE DISPERSION INDICES

To understand the basic properties of eigenvalue dispersion indices, we used a series of computer simulations. Our initial experiments were performed using a set of random observations drawn from a multivariate normal distribution defined as: N(0,Σ=I). This was equivalent to generating datasets of independent traits (e.g., linear measurements that are uncorrelated), or for the case of geometric morphometric data, generating shapes around the mean based on an isotropic model of landmark variation (Dryden & Mardia, 1998). The expectation (Adams, 2016; Adams & Collyer, 2016) was that under these conditions, reliable estimators of the magnitude of integration should remain stable over a wide range of both sample size (*N*) and the number of variables (*p*). Here, we examined six eigenvalue dispersion indices, which are enumerated in Table 1: (1) the integration coefficient of variation (ICV: Shirai & Marroig 2010), (2) the variance of the eigenvalues (VE: Wagner 1984), (3) the relative eigenvalue variance ($V_{rel}$: Pavlicev et al. 2009), (4) eigenvalue tightness (*T*_1_: Van Valen 1974), (5) the proportion of maximal variance (*T*_2_: Van Valen 1974), and (6) the relative eigenvalue dispersion ($D_{r}$: O'Keefe et al. 2022). Our goal was to determine which of these eigenvalue dispersion indices remained stable under the above conditions.

**Table 1:** Summary of eigenvalue dispersion indices examined in this article. Sources represent one primary reference where the index is described and defined. For all equations, λ represent the eigenvalues, *p* is the number of trait dimensions, and $R_{e}$ is the effective rank of the matrix (as defined in O'Keefe et al., 2022)

Index	Equation	Source
ICV	$\frac{\sigma_{\lambda }}{\bar{\lambda }}$	Shirai and Marroig, 2010
VE	$\frac{\sum {\left(\lambda_{i}-\bar{\lambda }\right)}^{2}}{p}$	Wagner, 1984
$V_{rel}$	$\frac{\sum {\left(\lambda_{i}-\bar{\lambda }\right)}^{2}}{p\left(p-1\right)\bar{\lambda }{}^{2}}$	Pavlicev et al. 2009
*T*_1_	$1-\frac{\sum \sqrt{\lambda_{i}}}{p\sqrt{\lambda_{1}}}$	van Valen, 1974
*T*_2_	$1-\frac{\sum \lambda_{i}}{p(\lambda_{1})}$	van Valen, 1974
$D_{r}$	$\frac{\sqrt[{2R_{e}}]{\prod \lambda_{R_{e}}}}{\sqrt{1/\pi R_{e}}}$	O'Keefe et al., 2022

#### Simulation Protocol

Simulated datasets were obtained by drawing *N* independent observations from a multivariate normal distribution, N(0,Σ), where Σ was a p×p identity matrix (e.g., Adams 2016; Adams & Collyer 2016; Watanabe 2022). For one set of simulations, we generated 1000 datasets with N=200 observations, at each of 17 different levels of variable number p=(5,20,35,⋯,250). Likewise, in a second set of simulations we obtained 1000 datasets with p=30 variables at each of 17 levels of sample size: N=(5,20,35,⋯,250). For each dataset, the trait covariance matrix was calculated, from which we obtained the corresponding set of eigenvalues. These were then used to calculate each of the six eigenvalue dispersion indices described above (Table 1). Eigenvalue dispersion indices were calculated using only the nontrivial dimensions of each dataset (i.e, where λ>0). This was in accord with recent work (O'Keefe et al., 2022) suggesting that rank‐deficient data dimensions should be excluded from the calculation of eigenvalue dispersion indices when estimating the magnitude of integration (see below for additional discussion). Additionally, a series of sampling experiments (Supporting Information) revealed that when rank‐deficient dimensions are included, estimates can be highly biased and misleading. Simulation code is found in the Supporting Information.

#### Results

**Figure 2:**
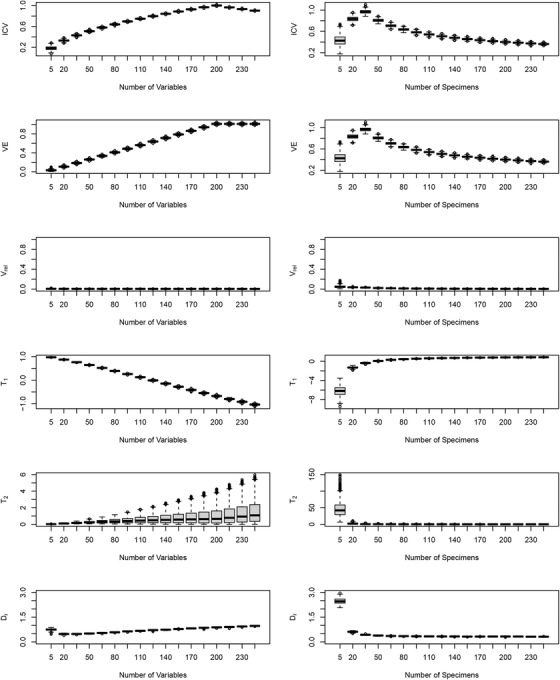
Patterns of eigenvalue dispersion indices across values of *N* and *p*, under the null hypothesis of no a priori association among variables. Eigenvalue dispersino indices are found in Table 1.

As is clear from Figure 2, most eigenvalue dispersion indices did not remain constant across differing levels of sample size or the number of variables. Instead, some increased as the number of variables (*p*) increased (see Pavlicev et al. 2009 for a verbal description of this for VE), while others decreased with increasing *p*. Additionally, most indices displayed a decrease with increasing sample size (*N*), though several first increased until N=p, and then decreased at higher *N*. Taken together, these results indicated that under scenarios where input levels of trait covariation were minimal, most eigenvalue dispersion indices did not remain stable. Instead, most varied with both *N* and *p*, implying these indices did not purely measure patterns of integration. Rather, they confounded integration with trends generated by both sample size and the number of variables. As a consequence, treating values based on these indices as estimates of the magnitude of integration is ill‐advised, and direct statistical comparisons of them across datasets are uninformative.

The one exception to this pattern was the relative eigenvalue variance, $V_{rel}$ (Fig. 2). Here, and as found previously by Watanabe (2022), it was observed that values of $V_{rel}$ remained stable across a wide range of both sample size and the number of variables. Further, the values obtained were close to zero, which corresponded with the input condition used in the simulations (that of no trait covariation). Therefore, in contrast to all other eigenvalue dispersion indices, $V_{rel}$ did accurately characterize input levels of covariation under these conditions.

### ADDITIONAL PROPERTIES OF $V_{rel}$

Based on the findings above, we evaluated several additional properties of $V_{rel}$ to determine whether it was a suitable candidate metric for estimating levels of integration, and whether it may used to statistically compare the magnitude of integration across datasets. Similar to Watanabe (2022), the first set of simulations investigated the efficacy of $V_{rel}$ to estimate known input levels of trait covariation. The second set of simulations investigated attributes of the sampling distribution of $V_{rel}$ across a range of input levels of covariation for the same *N* and *p*.

#### Simulation Protocol

The first set of simulations were performed using the procedure described in the previous section. Briefly, for N=128 across levels of p=(16,32,64,128,256), and for p=32 across levels of N=(16,32,64,128,256), 1000 datasets were generated by drawing *N* independent observations from a multivariate normal distribution, N(0,Σ). Here, Σ was a p×p trait covariance matrix which represented differing input levels of covariation: $V_{rel}=\left(0.1,0.3,0.6,0.9\right)$. To accomplish this, input covariance matrices were obtained following the computational procedures of Watanabe (2022). Specifically, Σ was defined as a diagonal matrix whose diagonal elements contained a set of descending eigenvalues that returned the desired $V_{rel}$ (code found in the Supplemental Information). Next, for each simulated dataset, eigenvalues were obtained from the trait covariance matrix, and the nontrivial eigenvalues were used to calculate $V_{rel}$. For each *N* and *p* combination, the mean and variance of $V_{rel}$ estimates were then obtained.

For the second set of simulations, we generated 2500 datasets of N=128 and p=32, at differing input levels of covariation: $V_{rel}=\left(0.1,0.3,0.5,0.7,0.9\right)$. For each dataset, $V_{rel}$ was obtained for the set of nontrivial eigenvalues obtained from the trait covariance matrix, and the sampling distributions at each input level of integration were generated. From these, the skewness and variance were observed. Additionally, to evaluate how the variance of $V_{rel}$ changed across its range, we repeated the simulation procedure above (with N=128 and p=32) at various input levels of covariation that spanned the range of possible input values ($V_{rel}=0.00\to 1.0$). At each input level, the variance of $V_{rel}$ was obtained from the corresponding sampling distribution. Simulation code for both procedures is found in the Supporting Information.

#### Results

**Figure 3:**
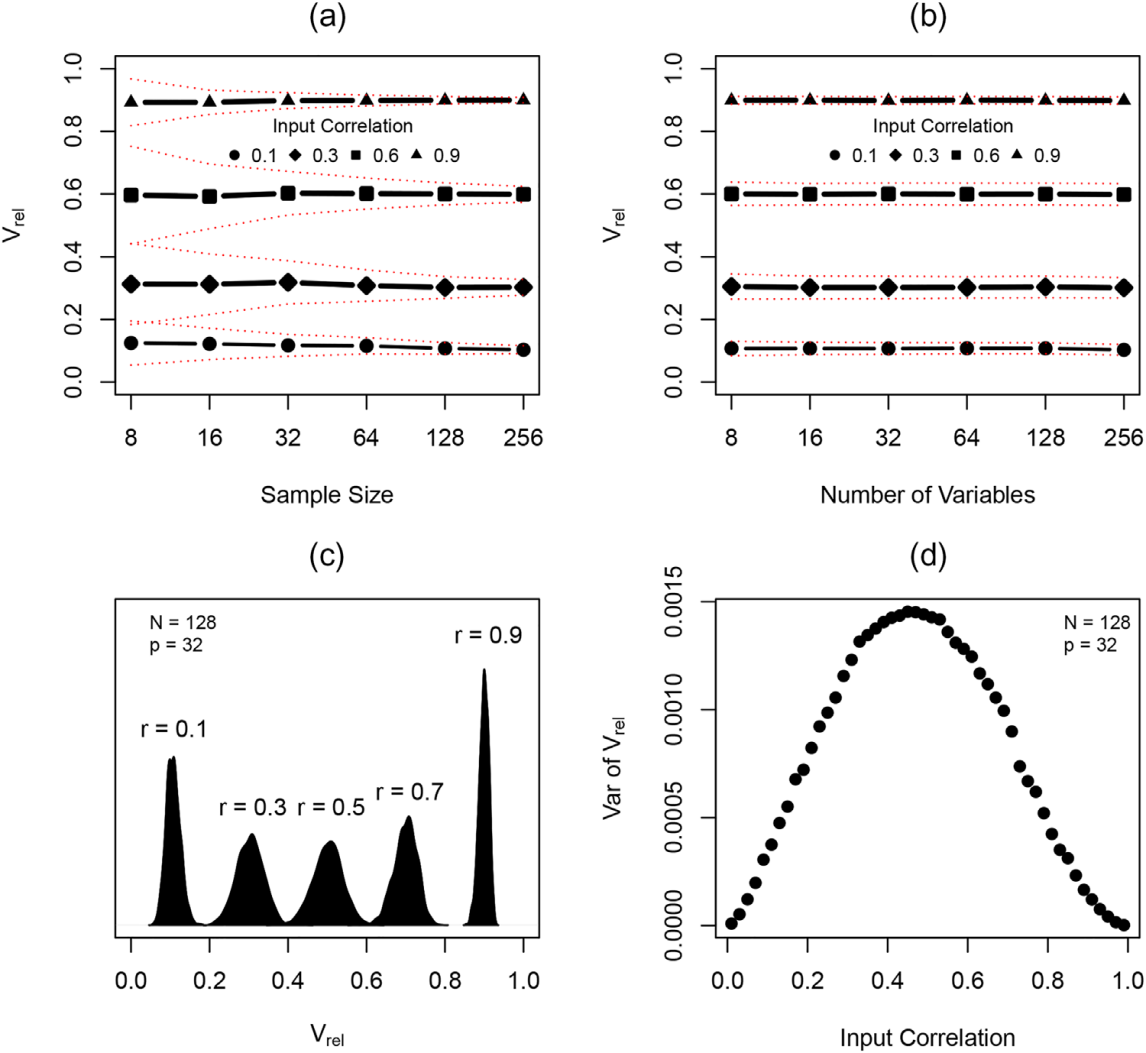
Simulation results estimating levels of integration based on the relative eigenvalue variance, $V_{rel}$. (a) Results across differing numbers of variables *p* at each of four different input correlation levels. (b) Results across differing numbers of specimens at each of four different input correlation levels. (c) Sampling distributions for $V_{rel}$ for the same *N* and *p* at differing levels of input correlation. (d) Variance in sampling distributions across a range of input correlation levels. Red dotted lines denote 95% confidence intervals.

At each input level of trait covariation, values of $V_{rel}$ remained constant across differing levels of sample size and across differing numbers of variables (Fig. 3a and b). This confirmed earlier findings of Watanabe (2022). As expected, the variance in $V_{rel}$ decreased with increasing sample size, conforming with predictions from parametric theory (Fig. 3a). Both of these properties suggested that $V_{rel}$ reliably estimates known input levels of covariation, and may thus be a suitable metric for representing the degree of integration in phenotypic datasets (as per Watanabe 2022). However, a visual inspection of confidence intervals (Fig. 2a and B: red lines) across input levels of covariation for the same *N* or *p* implied that variation was broader at intermediate input levels of covariation. Our second set of simulations investigated this pattern further.

When the sampling distributions of $V_{rel}$ were investigated across input levels of covariation (Fig. 3c), we found that they were relatively normal at intermediate values of $V_{rel}$. However, at extreme input values (i.e., $V_{rel}\approx 0$ & 1), the sampling distributions of $V_{rel}$ became more skewed (Fig. 3c). More importantly, we found that the sampling variance was not constant, but was largest at intermediate values of $V_{rel}$ and decreased at more extreme input levels; confirming our visual inspection of confidence intervals in Fig. 3a,b. An explicit quantification of the variance of the sampling distributions affirmed this observation (Fig. 3d), revealing that variance was smallest at the extreme values ($V_{rel}\approx 0$ & 1), and was highest at intermediate values ($V_{rel}\approx 0.5$). Together, these observations complicate direct statistical comparisons of $V_{rel}$, as they indicate that the sampling distribution of $V_{rel}$ can be non‐normal, and that the standard error of $V_{rel}$ does not remain constant across its range. As such, an alternative testing procedure for evaluating levels of covariation, and comparing its magnitude across taxa, is required.

### AN EFFECT SIZE FOR $V_{rel}$

As a solution to these issues, we contend that $V_{rel}$ must be converted to a standardized effect size for subsequent statistical evaluation. The logic underlying this suggestion is motivated by the properties of the bivariate correlation coefficient, ρ. As with $V_{rel}$, the sampling distribution of ρ becomes more non‐normal and skewed towards its extreme values (i.e,. ρ≈±1: see Fisher 1921). Likewise, the variance of ρ is not constant across its range, but is smallest toward its extreme values and highest at intermediate values: identical to the pattern found for $V_{rel}$ (Fig. 3d). Because of these sampling distribution properties, Fisher (1915) recognized that direct statistical comparisons of ρ were not useful, and instead proposed a transformation to a *Z*‐score; which rendered its distribution normal, and its standard error constant (Fisher, 1915, 1921, 1928). These effect sizes can then be used to make inferences regarding whether the correlation was stronger in one sample as compared with another. In light of the similar properties displayed by the sampling distribution of $V_{rel}$ (Fig. 3c,d), we suggest that the transformation of $V_{rel}$ to a standardized effect size via Fisher's *Z*‐transformation (Fisher, 1915) will alleviate the same issues for this statistic. The procedure is as follows.

First, one calculates $V_{rel}$ from the set of *p* nontrivial eigenvalues of the trait covariance matrix:

(1) $$\begin{array}{c} V_{rel}=\frac{\sum {\left(\lambda_{i}-\bar{\lambda }\right)}^{2}}{p\left(p-1\right)\bar{\lambda }{}^{2}} \end{array}$$

Next, a linear transformation is applied to $V_{rel}$, such that its range (originally 0 → 1) is transformed to −1→+1; matching that of ρ:

(2) $$\begin{array}{c} V_{rel}^{*}=2V_{rel}-1 \end{array}$$

Finally, $V_{rel}^{*}$ is converted to a standardized effect size using Fisher's *Z*‐transformation (Fisher, 1915):

(3) $$\begin{array}{c} Z_{Vrel}=\frac{1}{2}ln\left(\frac{1+V_{rel}^{*}}{1-V_{rel}^{*}}\right) \end{array}$$

**Figure 4:**
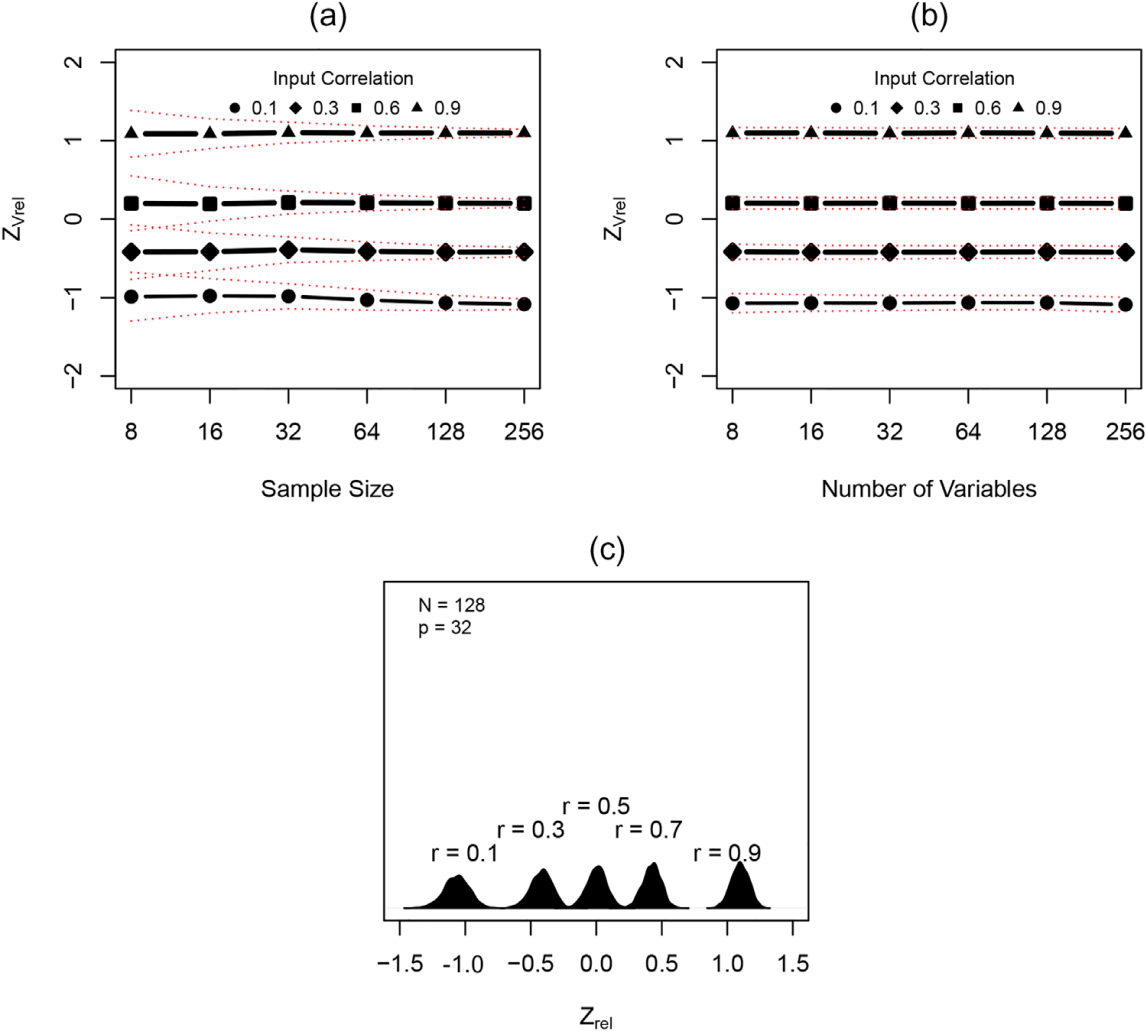
Simulation results estimating levels of integration based on the effect size, $Z_{Vrel}$. (a) Results across differing numbers of variables *p* at each of four different input correlation levels. (b) Results across differing numbers of specimens at each of four different input correlation levels. (c) Sampling distributions for $Z_{Vrel}$ for the same *N* and *p* at differing levels of input correlation. Red dotted lines denote 95% confidence intervals.

Figure 4 displays the outcome of converting $V_{rel}$ to $Z_{Vrel}$ for the simulated datasets examined previously. First, it can be seen that $Z_{Vrel}$ remained constant across both *N* and *p*, implying that this desirable property of $V_{rel}$ was retained in its corresponding effect size (Fig. 4a and b). Second, and unlike the patterns for $V_{rel}$, variation in $Z_{Vrel}$ across differing input levels of trait covariation was now relatively constant, as seen by comparing the confidence intervals of $Z_{Vrel}$ for the same *N* or *p* (vertical comparisons through Fig. 4a and b). This important property may also be seen in Fig. 4c, where transforming $V_{rel}$ to $Z_{Vrel}$ has rendered the sampling distributions of $Z_{Vrel}$ normal across the range of $V_{rel}$, and the variance in the sampling distribution relatively constant (Fig. 4c). Thus, as was observed with ρ, use of Fisher's *Z*‐transformation serves as a proper variance stabilizing transformation of $V_{rel}$. Further, the conversion of $V_{rel}$ to $Z_{Vrel}$ provides an effect size that reliably measures the magnitude of integration, which may subsequently be used as the basis for formal statistical comparisons of the strength of integration across datasets.

### HYPOTHESIS TESTING PROCEDURES

Once standardized effect sizes ($Z_{Vrel}$) have been estimated, one may test explicit hypotheses regarding the strength of morphological integration between datasets. For eigenvalue dispersion indices, such evaluations have previously been accomplished via bootstrapping procedures that establish confidence intervals for subsequent statistical hypothesis testing (e.g., Lewton 2012; Conaway et al. 2018; O'Keefe et al. 2022). However, because the sampling distributions for $Z_{Vrel}$ are approximately normal, an alternative approach is available. Specifically, the effect sizes ($Z_{Vrel}$) for two samples can be directly compared in a hypothesis test by finding the two‐sample test statistic:

(4) $$\begin{array}{c} {\hat{Z}}_{12}=\frac{|Z_{1}-Z_{2}|}{\sqrt{\sigma_{Z_{1}}^{2}+\sigma_{Z_{2}}^{2}}} \end{array}$$

Here, the effect sizes for each sample are found as: $Z_{1}=Z_{Vrel_{1}}$ and $Z_{2}=Z_{Vrel_{2}}$, and their standard errors are $\sigma_{Z_{obs_{1}}}=\frac{1}{\sqrt{N_{1}-3}}$ and $\sigma_{Z_{obs_{2}}}=\frac{1}{\sqrt{N_{2}-3}}$ respectively (for an empirical demonstration that $\sigma_{Z_{obs}}\approx \frac{1}{\sqrt{N-3}}$, see Supporting Information). With this statistic, the probability of ${\hat{Z}}_{12}$ under the null hypothesis of equal magnitudes of integration can then be estimated from a standard normal distribution (for similar procedures see: Adams & Collyer 2016, 2019a).

Additionally, because, the range of $Z_{Vrel}\approx \pm 3$ while the range of $V_{rel}=0\to 1$, it is natural to re‐express $Z_{Vrel}$ on a scale containing a lower bound of 0. This may be achieved as:

(5) $$\begin{array}{c} ZR=Z_{Vrel}+\left|Z_{0}\right| \end{array}$$

where $Z_{Vrel}$ represents the observed value, and *Z*_0_ is the *Z*‐score of $V_{rel}$ for independent variables. Importantly, this value is not the *Z*‐transformation of $V_{rel}=0$. Rather, it is the *Z*‐transformation of the *expected value* of $V_{rel}$, given the observed sample size and number of variables. Following (Watanabe (2022), eq. 24), for centered data this value is found as: $V_{rel_{0}}=\left(p+2\right)/\left(p*\left(N-1\right)+2\right)$. $V_{rel_{0}}$ is then converted to an effect size using equations 2 & 3 above. With this conversion, ZR ranges from zero (no trait covariation) to positive values (increasing levels of trait covariation), and thus expresses the magnitude of integration in a manner analogous to that of $V_{rel}$. As such, ZR serves as a useful heuristic to aid in the interpretation of the magnitude of integration in phenotypic datasets.

### STATISTICAL PERFORMANCE OF ${\hat{Z}}_{12}$

To evaluate the statistical performance of ${\hat{Z}}_{12}$, we performed two sets of simulation experiments. The first set of simulations evaluated the type I and type II error, as well as model misspecification rates. Here, we generated 1000 datasets of N=128 and p=16 for each of five different “populations,” which contained differing levels of trait covariation (none, low, or high). As in the previous simulations, *N* independent observations were drawn from a multivariate normal distribution, N(0,Σ), where Σ was a p×p trait covariance matrix representing the input level of covariation for that population: $V_{rel}=\left(0.0,0.0,0.3,0.6,0.6\right)$. As above, input covariance matrices were obtained following the computational procedures of Watanabe (2022). Next, $V_{rel}$ and $Z_{Vrel}$ were obtained for each population, and covariation levels were compared using ${\hat{Z}}_{12}$. The proportion of times the hypothesis of equal integration between pairs of populations was rejected was treated as a measure of type I error ($V_{rel}=0.0$ vs. $V_{rel}=0.0$), model misspecification rate ($V_{rel}=0.6$ vs. $V_{rel}=0.6$), or statistical power (1 ‐ type II error: $V_{rel}=0.3$ vs. $V_{rel}=0.6$ & $V_{rel}=0.0$ vs. $V_{rel}=0.6$). This procedure was repeated 100 times, and the mean outcome obtained.

The second set of simulations evaluated type I error and statistical power of ${\hat{Z}}_{12}$. Here, 1000 datasets were generated for p=10 traits for multiple levels of sample size N=(16,32,64,128,256), using the same procedure. Datasets were generated at several input levels of trait covariation: $V_{rel}=\left(0.0,0.0,0.2,0.4,0.6,0.8,1.0\right)$. As above, input covariance matrices were obtained following the computational procedures of Watanabe (2022). Next, $V_{rel}$ and $Z_{Vrel}$ were obtained for each dataset, and covariation levels for the first dataset ($V_{rel}=0.0$) were compared the rest using ${\hat{Z}}_{12}$. The proportion of times the hypothesis of equal covariation was rejected was treated as an estimate of type I error rate or statistical power, depending upon initial conditions. Simulation code for both procedures is found in the Supporting Information.

#### Results

**Figure 5:**
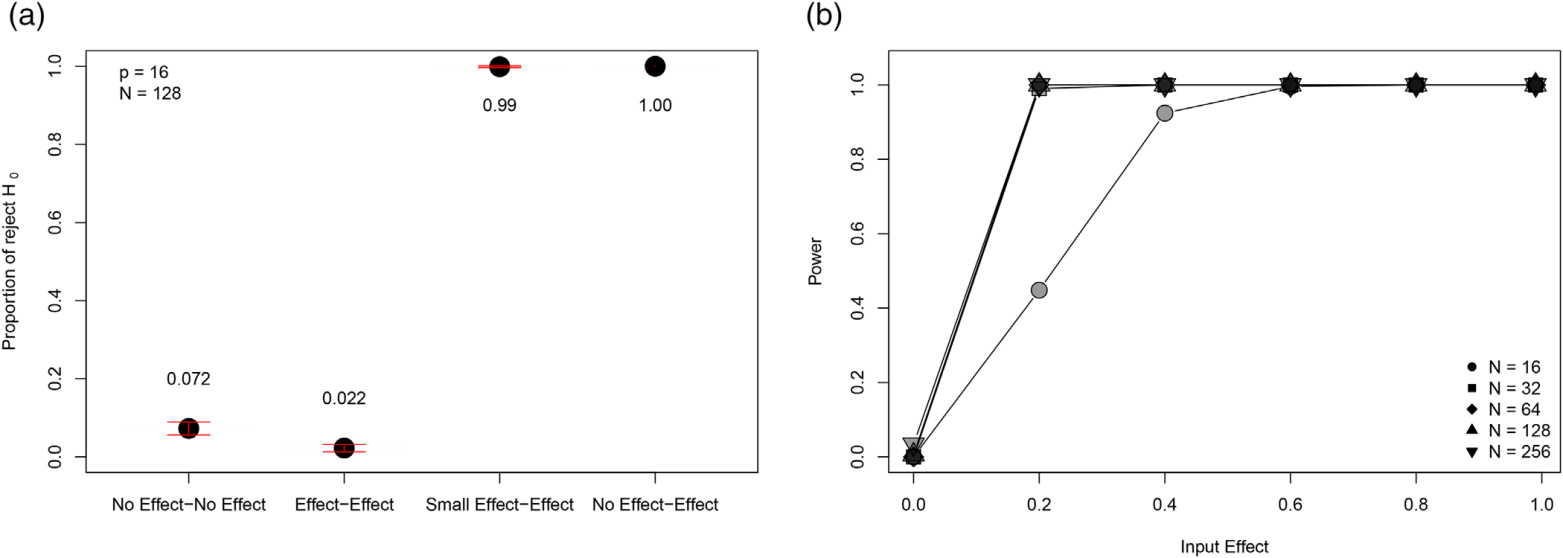
Simulation results showing: (a) Proportion of times the null hypothesis was rejected in 200 runs of 200 comparisons of morphological integration among datasets containing no integration (no effect), small levels of integration (small effect), and large levels of integration (large effect). Means with 95% confidence limits are shown, (b) Power curves for two‐sample tests comparing effect sizes of integration. The different curves correspond to simulations at differing levels of sample size.

As shown in Figure 5, two‐sample tests based on ${\hat{Z}}_{12}$ displayed appropriate statistical properties. First, the type I error of tests based on ${\hat{Z}}_{12}$ were at nominal levels for “no effect” comparisons (Fig. 5a), and across a range of sample sizes (Fig. 5b). Second, when the degree of covariation was similar in both datasets (i.e., “effect vs. effect”), levels of model misspecification rates were similarly low (Fig. 5b). However, when there were differences in the strength of covariation between datasets, these were detected at high frequency, indicating that type II error rates of ${\hat{Z}}_{12}$ were low and statistical power was high (Fig. 5a). Further, formal power analyses revealed that tests based on ${\hat{Z}}_{12}$ were capable of detecting small differences in the strength of trait covariation, and this ability improved with increasing sample size (Fig. 5b). Overall these simulation results revealed that tests comparing the strength of trait covariation between datasets based on ${\hat{Z}}_{12}$ were capable of detecting differences when they were present, and displayed low misspecification rates when levels of trait covariation were similar between datasets.

### A BIOLOGICAL EXAMPLE

To illustrate the method described above, we conducted a comparison of levels of integration of the os coxa (one half of the bilaterally symmetrical pelvis) across six primate species. In primates, variation in pelvic morphology is largely driven by selection on locomotor performance and function (Lewton, 2012). For example, in quadrupedal primates, the cranio‐caudal dimensions of the iliac blade are expanded to enhance stability of the lower back, which is in contrast to what is observed in primates with a more upright posture for whom lower back flexibility is vital (Robinson, 1972; Lovejoy et al., 1973). Second, the morphological moment arm of the ischium provides an indication of the mechanical advantage of the hamstring muscles in quadrupedal taxa (Fleagle & Anapol, 1992; Lovejoy et al., 1973; Robinson, 1972; Stern & Susman, 1983), while for humans and other hominins with a striding gait, this mechanical advantage is significantly reduced (Lovejoy et al., 1973). Finally, the constraints of childbirth in humans adds to the unique locomotory challenges of bipeds, as a highly encephalized neonate imparts distinct obstetric demands on the human pelvis that make childbirth uncommonly complex and treacherous (Schultz, 1949; DeSilva, 2011; Grabowski, 2013). These complex interactions suggest that pelvic morphology is both relatively conserved and highly integrated in most anthropoid primates; with the notable exception of the apes, and modern humans in particular. Several lines of evidence suggest that humans and other great apes display lower levels of integration of the pelvis as compared with other primates (see e.g., Young et al. 2010; Grabowski et al. 2011; Grabowski 2013; Lewton 2015). Further, quadrupedal cercopithecoid (non‐ape) primates have been shown to have higher integration among homologous limb elements than apes, likely in order to ensure that proper limb proportions are maintained for quadrupedal locomotion (Hallgrimsson et al., 2002; Young and Hallgrimsson, 2005; Young et al., 2010). Lower integration of apes relative to cercopithecoids has been shown also in the axial skeleton, suggesting that this pattern may persist throughout the postcranium (Jung et al., 2021). Therefore, given the link between primate pelvic morphology and locomotion, and the previously documented differences in integration between apes and monkeys, it follows that humans and other apes may display lower levels of integration in the pelvis when compared to cercopithecoids, as has been shown previously (Grabowski et al., 2011; Grabowski, 2013).

**Figure 6:**
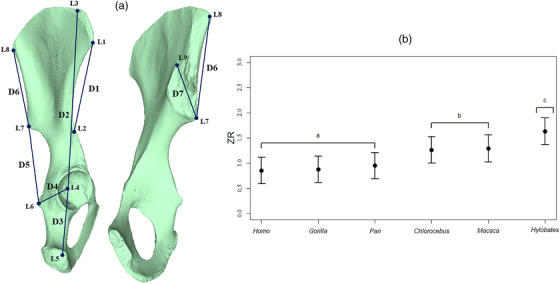
(a) Set of seven linear measurements used to characterize integration in the hominin pelvis. (b) Multivariate effect sizes and their 95% confidence intervals for each of six primate species.

To evaluate this hypothesis, we quantified levels of trait covariation in a set of seven linear distance measurements that spanned the functionally‐relevant regions of the os coxa (Fig. 6a). Each measure was chosen because of their hypothesized biomechanical role in primate locomotory function (see Grabowski et al. 2011). The data were collected as follows. First, an HDI‐120 surface scanner was used to capture 3D surface scans of non‐pathological, adult individuals (adult status determined by the eruption of the third molar and/or complete fusion of all postcranial epiphyses). Specimens were obtained from museum collections (American Museum of Natural History, Field Museum of Natural History, Museum of Comparative Zoology, Cleveland Museum of Natural History, Smithsonian National Museum of Natural History, and Univ. Buffalo Primate Skeletal Collection), with approximately an equal number of males and females per species. Sample sizes were also similar among species (*Homo sapiens*: N=60, *Hylobates lar*: N=57, *Chlorocebus pygerythrus*: N=60, *Macaca fascicularis*: N=56, *Gorilla gorilla*: N=59, and *Pan troglodytes*: N=60). Next, from each surface scan, the locations of eight three‐dimensional landmarks (Fig. 6a) were digitized using the program Landmark (Wiley et al., 2005). Landmark configurations were then subjected to a Generalized Procrustes Analysis (Rohlf & Slice, 1990) to align the specimens to a common coordinate system, and remove the effects of size. The set of seven linear distance measures (Fig. 6a) were then derived from the Procrustes‐aligned coordinates. Additionally, because the species differed substantially in overall size, and because size‐based allometry can contribute significantly to levels of integration (Bookstein et al., 2003; Klingenberg, 2013, 2014; Mitteroecker et al., 2005), we multiplied the Procrustes coordinates by size, and then calculated a second set of linear distance measures from these size‐incorporated coordinates. Thus, the second set of distance measures included variation in both size and shape.

Using each set of linear distance measures, we calculated the trait covariance matrix for each species (Σ) and estimated levels of integration as $V_{rel}$ using equation 1 above, calculated using the nontrivial dimensions of Σ. The $V_{rel}$ for all species were then converted to standardized effect sizes, $Z_{Vrel}$ using equations 2 and 3, so that the magnitude of integration in each species was represented on a common scale. We then determined whether the strength of integration differed among species, using the two‐sample *Z*‐score described above (Eq. 4). Based on the biomechanical considerations described above, we predicted that levels of integration would be significantly greater in the two cercopithecoid taxa (*Chlorocebus* and *Macaca*) as compared with the hominoid taxa. Finally and for comparison, we calculated $V_{rel}$ for the size‐standardized data with both the trivial and information‐deficient dimensions removed, where the latter were determined by information‐theory (i.e., those dimensions containing small, but non‐informative variation: see O'Keefe et al. 2022). All analyses were performed in R, using functions written by the authors (found in the Supplemental Information), and provided in the R‐package geomorph (Adams et al., 2022; Baken et al., 2021).

#### Results

When variation in both size and shape was examined, we found that all species displayed very high levels of integration in their pelvic dimensions ($V_{rel}=0.77\to 0.83$). By contrast, when differences in size were accounted for, levels of integration in the size‐adjusted traits dropped substantially ($V_{rel}=0.11\to 0.38$), although the rank‐order of values across species was consistent in both datasets. The comparison of size‐adjusted and size‐unadjusted values indicated that size effects and intraspecific allometry contributed substantially to overall levels of integration in these taxa. Given the body size differences among species, this result was not surprising. Subsequent comparisons of size‐standardized measurements revealed that the strength of integration in pelvic dimensions was not consistent across species, and our two‐sample test comparing effect sizes (*Z*‐scores) revealed that some species displayed significantly greater levels of morphological integration as compared with others (Fig. 6b, Table 2). In particular, we found that the cercopithecoid taxa (*Chlorocebus* and *Macaca*) displayed higher levels of integration as compared with several homonoid taxa (*Homo* and *Gorilla*); in accord with previous hypotheses. Somewhat surprisingly, one hominoid taxon, *Hylobates*, displayed the greatest levels of integration in their size‐adjusted traits, and was found to be significantly greater than patterns exhibited in the other hominoids (Fig. 6b, Table 2). This pattern differs from what was expected based on prior work on primate limb elements (Hallgrimsson et al., 2002; Young and Hallgrimsson, 2005), for example, Young et al. (2010). Finally, there was a near‐perfect correlation (ρ=0.96) between the set of $V_{rel}$ obtained using the nontrivial dimensions and those obtained when the information‐deficient dimensions removed, indicating that for this example, the small, but non‐zero dimensions had little effect on the empirical results. Overall, when viewed in light of the biomechanical predictions above, our data provide partial support for the hypothesis that when size is accounted for, hominoids present lower levels of integration in their pelvic morphology, relative to the cercopithecoid taxa. Thus, our findings are consistent with the biomechanical prediction that changes in locomotory mode have contributed to shifts in levels of morphological integration in *Homo* and *Gorilla* (Grabowski et al., 2011; Grabowski, 2013; Lewton, 2012, 2015).

**Table 2:** Pairwise tests (${\hat{Z}}_{12}$) comparing the strength of integration between species (above diagonal), and the corresponding significance level (below diagonal). Significant values are denoted with *

	Macaca	Chlorocebus	Pan	Homo	Hylobates	Gorilla
*Macaca*	—	0.1634	1.7911	2.297	1.7555	2.1799
*Chlorocebus*	0.8702	—	1.6581	2.1734	1.9516	2.0545
*Pan*	0.0733	0.0973	—	0.5154	3.5871	0.4038
*Homo*	0.0216*	0.0297*	0.6063	—	4.0955	0.1093
*Hylobates*	0.0792	0.051	$<0.0001^{***}$	$<0.0001^{***}$	—	3.97
*Gorilla*	0.0293*	0.0399*	0.6864	0.913	$<0.0001^{***}$	—

## Discussion

A major interest in evolutionary biology is determining whether taxa display similar or different levels of morphological integration, and deciphering why such differences evolve. Accomplishing this task requires summary test measures that accurately characterize the magnitude of morphological integration, and statistical tools that facilitate comparisons across sets of taxa or traits. Unfortunately, quantitative comparisons of the strength of integration in a set of traits across datasets have been limited, in part because statistical tools to facilitate these comparisons have remained underdeveloped. In this article, we propose a standardized effect size for this purpose ($Z_{Vrel}$), based on the relative eigenvalue variance, $V_{rel}$ found from the trait covariance matrix. This effect size is capable of capturing the magnitude of integration in a sample, and may be used in a testing procedure for comparing the strength of integration between datasets. In developing these tools, our work has revealed several key insights that advance the quantitative study of morphological integration, and further our understanding of the evolution of phenotypic diversity.

First, our findings demonstrate that most of the eigenvalue dispersion indices proposed as metrics of morphological integration (Haber, 2011; O'Keefe et al., 2022; Pavlicev et al., 2009; Shirai & Marroig, 2010; Van Valen, 1974; Wagner, 1984) inaccurately characterize such patterns. Our simulations revealed that when input levels of trait covariation were minimal, most eigenvalue dispersion indices were sensitive to both sample size and the number of variables; varying widely with changes in both (Fig. 2). Because of this dependence on *N* and *p*, interpreting levels of trait covariation with these indices is challenging, as the same value may be obtained from samples with differing numbers of observations, differing numbers of variables, differing levels of covariation, or some combination of the three. Further, while tests of these measures could be envisioned based on bootstrapping methods (e.g., Lewton 2012; Conaway et al. 2018; O'Keefe et al. 2022) or simulation procedures such as random skewers (Shirai & Marroig, 2010) but see Rohlf (2017), these procedures are less general, and will still be restricted to cases where either *N* or *p* is constant across datasets. In contrast to these findings, our investigations revealed that values of $V_{rel}$ remained stable under these conditions, regardless of sample size or the number of variables. Likewise, and as shown by Watanabe (2022), this constancy was retained at varying input levels of trait covariation (Fig. 3). Together, these patterns revealed that unlike other eigenvalue dispersion measures, $V_{rel}$ serves as a reliable estimator of the magnitude of trait covariation, and thus the strength of integration, in phenotypic datasets.

Second, while stability across *N* and *p* implies that $V_{rel}$ may be a suitable estimator of the magnitude of integration, our simulations revealed that the properties of its sampling distribution preclude direct statistical comparisons of this measure across datasets. Specifically, our work demonstrated that as $V_{rel}$ approached its extreme values, the sampling distribution became more skewed, and its error variance was greatly reduced (Fig. 3). The latter in particular compromises statistical comparisons of $V_{rel}$, because it becomes increasingly challenging to accurately estimate confidence intervals as $V_{rel}$ approaches its limits (for a similar discussion see: Fisher 1915; Hotelling 1953). Thus, in contrast to the suggestion of Watanabe (2022), our findings indicate that comparisons of the strength of integration across datasets should “not” be accomplished via direct statistical tests on $V_{rel}$, but rather based on effect sizes obtained from $V_{rel}$.

As a measure of the strength of integration, we proposed a standardized effect size, $Z_{Vrel}$, based on the transformation of $V_{rel}$ via Fisher's *Z*‐transformation. We demonstrated that $Z_{Vrel}$ was stable across both *N* and *p*, and across varying input levels of trait covariation; retaining the desirable properties of $V_{rel}$. But in addition, we also showed that $Z_{Vrel}$ maintained a constant variance throughout its range (Fig. 4). Thus, the conversion of $V_{rel}$ to $Z_{Vrel}$ alleviated the challenges identified with attempts to use $V_{rel}$ as a test statistic. As such, the desirable properties of $Z_{Vrel}$ revealed above sanction its use as an effect size in statistical comparisons of the strength of integration across datasets, and our two‐sample test (equation 4) provisions a procedure for its implementation. As illustrated with our empirical example, this approach provides a reliable means of comparing the magnitude of integration among species (or sets of traits), thereby opening the door to future examinations of the strength of integration in other empirical systems. Therefore, our proposed effect size ($Z_{Vrel}$), and associated two‐sample test, contribute important new comparative tools to the evolutionary biologist's quantitative arsenal for the study of phenotypic diversity.

An important consideration when characterizing patterns of morphological integration is how to treat redundant dimensions: those that contain no variation, or those that contribute no additional information in terms of phenotypic variation and covariation. From an empirical perspective, there are three common reasons why redundancies may be present in morphometric datasets which are unrelated to morphological integration, yet result in covariance matrices that are rank‐deficient. First, when one variable is a perfect linear combination of others (e.g., A+B=C), deficient dimensions will arise. Second, when data‐wide standardizations are performed, such as translation, rotation, and scaling of configurations of landmarks in geometric morphometric analyses (Adams et al., 2004, 2013), mathematical redundancies will be generated, resulting in singular dimensions. Additionally, the use of sliding semilandmarks (Gunz et al., 2005) or symmetrical structures will further exacerbate the issue, and contribute additional redundant dimensions. Third, when there are more trait dimensions than observations (i.e., p>N), there will be at least p−(N−1) redundant dimensions (Legendre & Legendre, 2012). All of these cases are common in morphometric datasets, and stem from data acquisition decisions, rather than reflecting levels of morphological integration *per se*. It therefore follows that such redundancies in morphometric data should be accounted for prior to summarizing overall levels of integration, and that such summaries should be based only on the nontrivial dimensions of the dataset (for a related discussion see Hansen and Houle 2008).

In this study, we eliminated those dimensions that contributed no variation (i.e., λ=0), and demonstrated by simulation that when those dimensions were included, $V_{rel}$ displayed biased estimates (Supplemental Information). Similarly, recent work by O'Keefe et al. (2022) suggested that all rank‐deficient data dimensions—both those that contribute no variation, as well as those that contain small amounts of variation but do not provide additional information (as defined by an entropy‐based measure: see O'Keefe et al. 2022)—should be excluded from the calculation of phenotypic integration indices. While it is incontrovertibly the case that trivial dimensions (i.e., λ=0) contribute nothing to trait covariation and should be removed prior to estimating levels of integration, whether or not one should concomitantly exclude dimensions with small variation is less clear. Excluding the latter based on an entropy‐based definition of rank‐deficiency implicitly assumes that variation in dimensions with small eigenvalues are effectively noise. This need not be the case, although we are unaware of any study that has examined this issue. In our empirical example, removing dimensions with small variation had little effect on the biological conclusions when compared with removing only the trivial dimensions, though in another empirical example integration estimates varied considerably more (O'Keefe et al., 2022). We suggest that future simulation‐based studies examine the conditions under which the removal of both trivial and rank‐deficient dimensions is warranted. Finally, we emphasize that for studies of integration to be biologically meaningful, the set of traits under investigation must be in commensurate units and of similar scale. Otherwise, the distances between specimens, and the covariances between variables—the summary values upon which statements of integration are made—are uninterpretable (Adams and Collyer, 2019b; Huttegger & Mitteroecker, 2011).

The analytical advances forwarded in this study provide a quantitative means for characterizing the degree of trait covariation in phenotypic datasets, and comparing the strength of this integration among taxa. However, a related aspect of morphological integration that is not addressed by the current proposal is characterizing the manner in which traits are integrated. Theoretically, it is possible that two taxa may display similar levels of overall integration, yet the pairwise covariances among traits may differ. In this case, a subsequent investigation of the contributions of individual trait dimensions to levels of variation would prove fruitful, through scrutiny of the trait loadings along their respective principal axes (as found via eigenanalysis, factor analysis, singular‐value decomposition, or related procedure). Further, one may compare the manner in which traits covary between taxa by an assessment of the extent to which the direction of their principal axes coincide (Begin & Roff, 2003; Klingenberg & Leamy, 2001; Schluter, 1996). When viewed from this perspective, tests of the magnitude of integration and a description of the how traits covary are complementary, and provide a two‐stage procedure for examining such patterns. In this sense, interrogating trends of morphological integration may be viewed in a similar manner to approaches for evaluating the tempo and mode of phenotypic evolution (Simpson, 1944), or evaluating the magnitude and direction of phenotypic evolutionary trajectories (Adams & Collyer, 2009; Collyer & Adams, 2007). The methods proposed here provide one important component of this emerging analytical paradigm.

One important extension of the approach developed here is for quantifying patterns of evolutionary integration. For this application, integration is estimated from a set of observations (species means) that are not independent of one another, but rather whose values are expected to be correlated in a manner as described by their phylogenetic relationships (Felsenstein, 1985). In this case, $Z_{Vrel}$ must be estimated from the evolutionary covariance matrix, which represents the set of trait covariances conditioned on the phylogeny (see: Revell & Harmon 2008; Adams 2014b; Zelditch & Goswami 2021). Indeed, use of the evolutionary covariance matrix is common in phylogenetic comparative methods for multivariate data (e.g., Revell & Harmon 2008; Klingenberg & Marugán‐Lobón 2013; Adams 2014a; Adams & Felice 2014; Adams & Collyer 2018; Adams and Collyer 2019b; Clavel et al. 2019), and methods for evaluating evolutionary trends of the integration between modules have made explicit use of this matrix (Adams & Felice, 2014; Klingenberg & Marugán‐Lobón, 2013). Thus, the extension proposed here is in line with prior approaches which have accounted for phylogenetic non‐independence under a Brownian motion model of evolution. Otherwise, for macroevolutionary applications, the approach developed here remains fundamentally unaltered.

Finally, the methods advanced here add to a burgeoning body of biometric theory that leverages effect sizes for the comparison of the strength of biological signal in multivariate phenotypic datasets. Past work utilized permutation‐based empirical effect sizes for evaluating the capacity of factors in linear models to describe variation in multivariate datasets (Adams & Collyer, 2018; Collyer et al., 2015). Other methods compared the strength of phylogenetic signal as described by standardized effect sizes (Collyer et al., 2022). More directly related to the present study, analytical approaches have been developed to compare multivariate effect sizes that characterize and compare the strength of modularity in phenotypic datasets (Adams & Collyer, 2019a), as well as for comparing levels of integration between modular structures (Adams & Collyer, 2016). While these approaches provide important tools for the comparison of patterns of phenotypic covariation, both presuppose that modular structure is present in phenotypic datasets. Our method contributes to this theme, but provides an analytical tool that enables the ability to compare levels of integration across datasets without the a priori assumption of modularity. Thus when viewed in this light, the work presented here provides an important analytical tool for the study of trait covariation, as it facilitates the characterization and comparison of integration patterns where modular structure need not be imposed or required.

## AUTHOR CONTRIBUTIONS

M.A.C. and D.C.A. collaboratively developed the concept and contributed to all portions of this manuscript. All authors approve of the final product and are willingly accountable for any portion of the content.

Open access funding provided by the Iowa State University Library.

## CONFLICTS OF INTEREST

The authors declare no conflict of interest.

## DATA ARCHIVING

Data for empirical example is available on DRYAD (https://doi.org/10.5061/dryad.tb2rbp038). R‐scripts for simulation tests are found in the Supplemental Information.

Associate Editor: M. Pavlicev

Handling Editor: M. L. Zelditch

## Supporting information


**Figure 1**: (A) Emprical variance in ZV rel as estimated across simulated datasets for differing levels of sample size (N).
**Figure 2**: Simulation demonstrating an increase in estimates of Vrel with an increasing number of redundant dimensions, when using the trait covariance matrix as input.
**Figure 3**: Simulation demonstrating a decrease in estimates of Vrel with an increasing number of redundant dimensions, when using the trait correlation matrix as input.

Data S1

Data S1
