# Supplementary material for: An effect size for comparing the strength of morphological integration across studies
Source: Evolution. 2022 Aug 22;76(10):2244–59. doi: 10.1111/evo.14595 (PMC9804739; doi:10.1111/evo.14595)
Supplement: Supplementary file 1 — Figure 1: (A) Emprical variance in ZV rel as estimated across simulated datasets for differing levels of sample size (N). Figure 2: Simulation demonstrating an increase in estimates of Vrel with an increasing number of redundant dimensions, when using the trait covariance matrix as input. Figure 3: Simulation demonstrating a decrease in estimates of Vrel with an increasing number of redundant dimensions, when using the trait correlation matrix as input. [file EVO-76-2244-s002.pdf]

An Effect Size for Comparing the Strength of Morphological Integration Across Studies: Supporting Information

1: Empirical Estimate of the Variance of Z_{Vrel}

As described in the main article, the relative eigenvalue variance, V_{rel}, serves as a reliable index of the degree of integration in a set of phenotypic traits. However, because the sampling distribution of V_{rel} becomes more skewed towards its extreme values, and its variance changes with differing input levels of integration, it must first be transformed to an effect size for subsequent statistical evaluation. We developed a standardized effect size (Z_{Vrel}) for V_{rel}, based on Fisher's Z-transformation. We further proposed that $\sigma^2_Z = 1/(N-3)$ serves as an approximation of its variance. Here we use simulations to demonstrate that this approximation is generally appropriate for the variance of Z_{Vrel}.

Simulation Protocol

To empirically estimate the variance of Z_{Vrel}, we first generated a $p \times p$ covariance matrix $\mathbf{\Sigma}$, whose elements contained input values that would result in a $V_{rel} \approx 0.6$. The source code `WatanabeCov.r` (provided as supporting information) contains functions for obtaining this covariance matrix, following Watanabe (2022). Next we simulated multivariate datasets by drawing independent observations from a normal distribution of $\mathcal{N}(0, \mathbf{\Sigma})$. With this procedure we simulated 1000 multivariate datasets at each of 20 different levels of sample size: $N = (10, 35, 60, \ldots, 500)$. For each dataset, we then calculated the associated V_{rel}, found from the set of non-trivial eigenvalues (i.e., $\lambda > 0.0$) obtained from the trait covariance matrix. Following the main article, each V_{rel} was then linearly-transformed to the scale $(-1.0 \rightarrow 1.0)$ as: $V^*_{rel} = (2 * V_{rel}) - 1$. Next, the transformed values were converted to effect sizes using Fisher's Z-transformation: $\left(Z_{Vrel} = \frac{1}{2} ln\left(\frac{1+V^*_{rel}}{1-V^*_{rel}}\right)\right)$, and the variance across the 1000 effect sizes at each level of N was obtained. These values were then compared to values using the approximation: $\sigma^2_Z = 1/(N-3)$. Finally, the entire simulation was repeated at each of 25 levels of variable number: $p = (10, 20, 30, \ldots, 250)$, to determine whether the number of variables affected the expected variation. The variance of Fisher's Z for the bivariate correlation coefficient (ρ) was also estimated using an analogous procedure, to form a basis of comparison for using $\sigma^2_Z = 1/(N-3)$ for Z_{Vrel}.

Data Simulation Script

```
library(MASS)
source('WatanabeCov.r')

#ZR function
Z.Vrel <- function(x){
  if(length(dim(x))==3){
    x <- geomorph::two.d.array(x)
  }
  n <- nrow(x)
  if (!is.null(phy)) {
    phy.parts <- geomorph:::phylo.mat(x, phy)
    Ptrans <- phy.parts$D.mat %*% (diag(n) - matrix(1, n) %*%
                crossprod(matrix(1, n), phy.parts$invC)/sum(phy.parts$invC))
    x <- Ptrans %*% x
  }
```

```
  eig.obs <- eigen(cov(x))$values
  eig.obs <- eig.obs[which(zapsmall(eig.obs)>0)]
  p <- length(eig.obs)
  Vrel.exp <- ((2*((p+2)/((p*(n-1))+2)))-1)
  Re.obs <- var(eig.obs) / (mean(eig.obs)^2*p)
  Re.obs.trans <- ((2*Re.obs)-1)
  if(Re.obs.trans==1){Re.obs.trans=0.999}
  if(Re.obs.trans==-1){Re.obs.trans=-0.999}
  Z.obs <- 0.5*log((1+Re.obs.trans) / (1-Re.obs.trans))
  ZR <- Z.obs + abs(ZN)
  ZR.var <- 1/(n-3)
  out <- list(Re.obs = Re.obs, Z.obs = Z.obs, ZR = ZR, ZR.var = ZR.var)
  class(out) <- "rel.eig"
  out
}

#Simulate Variance of the ZR
n <- seq(10,500,25)
p <- seq(10,250,10)
nsets <- 1000

ZR.var <- lapply(1:length(n), function(x){
  cat("| N =", n[x], ":")
  lapply(1:length(p), function(i){
    dat <-lapply(1:nsets, function(j){
      Sigma <- GenCov(p[i], VR = 0.6, shape = "q-large")
      mvrnorm(n = n[x], mu = rep(0,p[i]), Sigma = Sigma)
    })
    cat(" p =", p[i], ",")
    VErel <- unlist(lapply(1:nsets, function(k) {
      Z.Vrel(dat[[k]])$ZR
    }))
  })
})
ZR.var2 <- simplify2array(ZR.var)

#Simulate Variance of the Bivariate Correlation
biv.dat <- lapply(1:length(n), function (x){
  z <- function(y){
    z <- 0.5*log((1+y) / (1-y))
  }
  dat <-lapply(1:nsets, function(j){
    Sigma <- matrix(0.6, 2, 2); diag(Sigma) <- 1
    mvrnorm(n = n[x], mu = rep(0,2), Sigma = Sigma)
  })
  corr <- lapply(1:nsets, function(k){
    cor.test(dat[[k]][,1],dat[[k]][,2])$estimate
  })
  zscore <- unlist(lapply(1:nsets, function(i){
    z(corr[[i]])
  }))
  vary <- var(zscore)
})
```

Simulation Results

As expected, the sampling variation of Z_{Vrel} decreased with increasing N (Fig. 1A). Patterns were largely consistent across values of p, implying that the sampling variation of Z_{Vrel} was relatively insensitive to variable number. $\sigma^2_Z = 1/(N-3)$ was found to be a reasonable approximation to the empirical variance at large N. It was noted that at small sample sizes, $\sigma^2_Z = 1/(N-3)$ overestimated the true variance slightly (Fig. 1A). This was in contrast to what is found with Fisher's Z for the correlation coefficient, where $\sigma^2_Z = 1/(N-3)$ matched empirical estimates. Note, however, that for Z_{Vrel}, a slight overestimate of its variance will result in statistical tests that are more conservative, as σ^2_Z is found in the denominator of both one-sample and two-sample tests (see main article).

Overall, these results revealed that $\sigma^2_Z = 1/(N-3)$ was a reasonable first approximation to the sampling variation of Z_{Vrel} across both N and p.

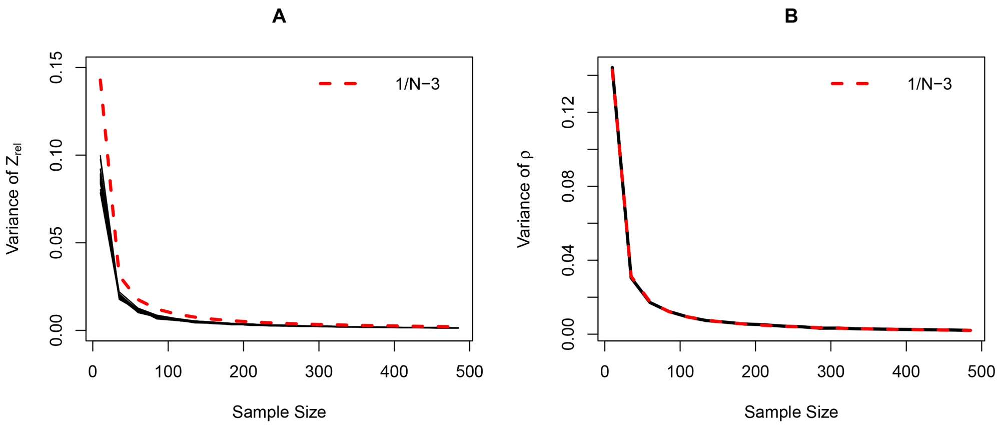

Figure 1: (A) Emprical variance in Z_{Vrel} as estimated across simulated datasets for differing levels of sample size (N). The different lines (black) correspond to different numbers of variables (p). The expected variance is shown in red. (B) Emprical variance in Z_ρ as estimated across simulated datasets for differing levels of sample size (N). The expected variance is shown in red.

2: Effect of Redundant Dimensions (Rank-Deficiency) on V_{rel}

As described in the main article, an important consideration when estimating the relative eigenvalue variance, V_{rel}, is how to treat those dimensions with zero variation (i.e., for covariance matrices, dimensions whose $\lambda = 0.0$). Recent work (O'Keefe et al. 2022) suggests that rank-deficient data dimensions should be excluded from the calculation of eigenvalue dispersion indices when estimating the magnitude of integration. Empirically, there are three primary reasons why redundancies exist in phenotypic datasets; resulting in covariance matrices that are rank-deficient:

- When $p > N$
- When one variable is a perfect linear combination of others (e.g., $A + B = C$)
- When data-wide standardizations are performed, resulting in linear dependencies (e.g., landmark configurations are translated, rotated, and scaled)

The consequence of any of these is that one or more data dimensions are rendered singular, and thus one or more eigenvalues of the covariance matrix will be equal to zero. With respect to summarizing integration, the question then is whether these trivial dimensions should be included.

We contend that for estimates of integration, calculations of eigenvalue dispersion indices such as V_{rel} should be based on only the *non-trivial dimensions* of the dataset. In this section, we provide a simple empirical demonstration that complements the suggestion of O'Keefe et al (2022), and shows that when redundant dimensions are retained, the resulting estimates of V_{rel} from covariance matrices are positively biased.

Simulation Protocol

To demonstrate the effect of redundant dimensions on estimates of V_{rel}, we simulated data under three different scenarios. The first contained many more observations than variables ($N >> p$), with $N = 500$ and $p = 30$. The second contained many more variables than observations ($p >> N$), with $N = 30$ and $p = 500$. The third contained an equal number of observations and variables ($N = p$) at small sample size, with $N = 8$ and $p = 8$. For each scenario we generated a $p \times p$ covariance matrix $\mathbf{\Sigma}$, whose elements contained input values that would result in a $V_{rel} \approx 0.6$. The source code `WatanabeCov.r` (provided as supporting information) contains functions for obtaining this covariance matrix, following Watanabe (2022). Next we simulated 1000 datasets for each scenario by drawing independent observations from a normal distribution of $\mathcal{N}(0, \mathbf{\Sigma})$, based on N and p as specified above. These represented the 'base' datasets for each scenario.

Using the base datasets, a series of rank-deficient datasets were then constructed, by adding one or more dimensions of trivial information to each matrix. This was accomplished by appending additional columns of 0 to each dataset ($p_0 = 4, 100, 400$). We then obtained the eigenvalues for the covariance matrices for all datasets, calculated its associated V_{rel}, and obtained mean values for each scenario and level of redundancy.

Finally, the first of these scenarios ($N >> p$) was repeated, simulating data with an input correlation matrix whose off-diagonal elements were $\rho \approx 0.6$). In this simulation, V_rel was estimated from the correlation matrix rather than the covariance matrix. Because the redundant dimensions contained no variation (and thus have no calculable covariance), those elements of the correlation matrix were replaced by zeros. All remaining calculations were repeated as above.

Data Simulation Script

```r
library(MASS)
source('WatanabeCov.r')

#Rel.Eig Function
Relve <- function(x){
  eig.obs <- eigen(cov(x))$values
  p <- length(eig.obs)
  Re.obs <- var(eig.obs) / (mean(eig.obs)^2*p)
  Re.obs
}

#Rel.Eig.Zap Function
Relve.zap <- function(x){
  eig.obs <- eigen(cov(x))$values
  eig.obs <- eig.obs[which(zapsmall(eig.obs)>0)]
  p <- length(eig.obs)
  Re.obs <- var(eig.obs) / (mean(eig.obs)^2*p)
  Re.obs
}

#Rel.Eig Correlation Function
Relve.cor <- function(x){
  R <- cor(x)
  R[is.na(R)] <- 0
  eig.obs <- eigen(R)$values
  p <- length(eig.obs)
  Re.obs <- var(eig.obs) / (mean(eig.obs)^2*p)
  Re.obs
}
```

```r
#Scenario 1: N>p
n <- 500
p <- 30

results.1 <- lapply(1:1000, function(i){
  Sigma <- GenCov(p, VR = 0.6, shape = "q-large")
  Y <- mvrnorm(n = n, mu = rep(0,p), Sigma = Sigma)
  Y2 <- cbind(Y,matrix(0, n, 4))
  Y3 <- cbind(Y,matrix(0, n, 100))
  Y4 <- cbind(Y,matrix(0, n, 400))
 res<- cbind(
    Relve(Y),
    Relve(Y2),
    Relve(Y3),
    Relve(Y4)
  )
})


#Scenario 2: p>N
n <- 30
p <- 500

results.2 <- lapply(1:1000, function(i){
  Sigma <- GenCov(p, VR = 0.6, shape = "q-large")
  Y <- mvrnorm(n = n, mu = rep(0,p), Sigma = Sigma)
  Y2 <- cbind(Y,matrix(0, n, 4))
  Y3 <- cbind(Y,matrix(0, n, 100))
  Y4 <- cbind(Y,matrix(0, n, 400))
  res<- cbind(
    Relve.zap(Y),
    Relve(Y),
    Relve(Y2),
    Relve(Y3),
    Relve(Y4)
  )
})


#Scenario 3: small N=p
n <- 8
p <- 8

results.3 <- lapply(1:1000, function(i){
  Sigma <- GenCov(p, VR = 0.6, shape = "q-large")
  Y <- mvrnorm(n = n, mu = rep(0,p), Sigma = Sigma)
  Y2 <- cbind(Y,matrix(0, n, 4))
  Y3 <- cbind(Y,matrix(0, n, 100))
  Y4 <- cbind(Y,matrix(0, n, 400))
  res<- cbind(
    Relve.zap(Y),
    Relve(Y),
    Relve(Y2),
```

```
    Relve(Y3),
    Relve(Y4)
  )
})

#Scenario 4: N>p with correlation matrix
n <- 500
p <- 30

results.cor <- lapply(1:1000, function(i){
  Sigma <- matrix(.6,p,p); diag(Sigma) <- 1
  Y <- mvrnorm(n = n, mu = rep(0,p), Sigma = Sigma)
  Y2 <- cbind(Y,matrix(0, n, 4))
  Y3 <- cbind(Y,matrix(0, n, 40))
  Y4 <- cbind(Y,matrix(0, n, 100))

  res<- cbind(
    Relve.cor(Y),
    Relve.cor(Y2),
    Relve.cor(Y3),
    Relve.cor(Y4)
  )
})
```

Simulation Results

As seen in Figure 2, when redundant dimensions were included in the calculation of V_{rel} based on covariance matrices, estimates of V_{rel} were always higher than the original value (when using the correlation matrix, V_{rel} decreased). Further, as the number of redundant dimensions increased, so too did the bias in V_{rel}. This pattern was most acute in datasets of small sample size (Fig. 2C).

Additionally, when the correlation matrix was used instead of the covariance matrix, the pattern was reversed. Here including more and more rank-deficient dimensions to the dataset resulted in a decrease in estimates of V_{rel} (Fig. 3). Here the pattern was more pronounced, and V_{rel} dropped precipitously and approached zero as the number of redundant dimensions increased. This pattern would incorrectly imply that there was no covariation between traits, when in fact it was due to having many redundant dimensions in the dataset.

Overall, this illustration demonstrates that retaining redundant dimensions in the dataset results in a bias in estimates of V_{rel}, and thus in the degree of integration among traits. Further, the direction of this bias depends upon whether the trait covariance matrix or correlation matrix is used. Either way, this demonstration provides empirical justification that redundant dimensions with zero variation must be excluded from calculations of eigenvalue dispersion indices, if those estimates are to be an accurate reflection of the actual patterns in the data (see also O'Keefe et al. 2022).

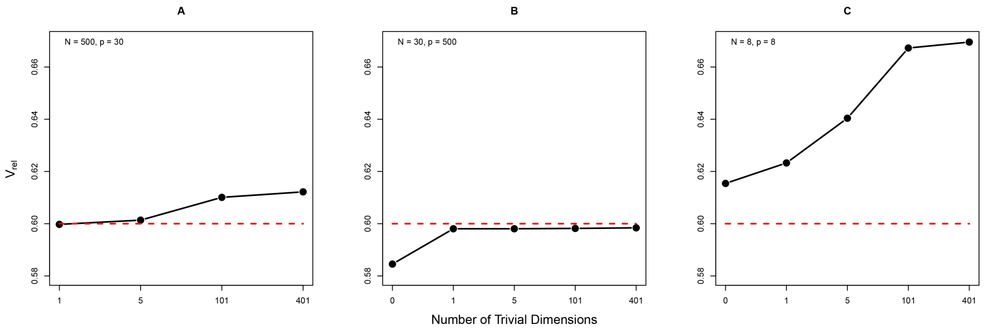

Figure 2: Simulation demonstrating an increase in estimates of V_{rel} with an increasing number of redundant dimensions, when using the trait covariance matrix as input. Values represent the mean across 1000 datasets under each condition.

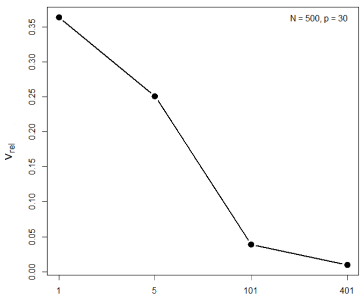

Figure 3: Simulation demonstrating a decrease in estimates of V_{rel} with an increasing number of redundant dimensions, when using the trait correlation matrix as input. Values represent the mean across 1000 datasets under each condition.

3: Simulation Scripts for Main Article

In this section, we provide all simulation scripts used to generate the primary results in the main article.

3.1: Comparison of Eigenvalue Dispersion Indices

In the main article, we compared the performance of six eigenvalue dispersion indices under the hypothesis of no integration (i.e., a random collection of variables). The following r-script was used to generate Figure 2.

```
#Integration Functions
eig.zap <- function(x){
  eig<- eigen(cov(x))$values
  eig
}
ICV <- function(eig) {
  eig<- eig[which(zapsmall(eig)>0)]
 ICV <- sd(eig)/mean(eig)
 ICV
}
VE <- function(eig){
  eig<- eig[which(zapsmall(eig)>0)]
```

```
  VE <- var(eig)/mean(eig)
  VE
}
RelVE <- function(eig){
  eig<- eig[which(zapsmall(eig)>0)]
  relvE <- var(eig)/ (mean(eig)^2 *length(eig ))
  relvE
}
T1 <- function(eig){
  eig<- eig[which(zapsmall(eig)>0)]
  Tight <- 1 - (sum(sqrt(eig))/length(eig)*sqrt(eig[1]))
  Tight
}
T2 <- function(eig){
  eig<- eig[which(zapsmall(eig)>0)]
  Tight <- 1 - ((sum(eig)/length(eig)*eig[1]))
  Tight
}
RelDisp <- function(eig){
  eig <- ifelse(eig==0,0.0001,eig)
  EffRk <- exp(-1*sum((log(eig / sum(eig)))*(eig / sum(eig)), na.rm=TRUE))
  SVG <- (prod(eig))^(1/length(eig))
  EffRkSGV<-(prod(eig[1:trunc(EffRk)])*eig[trunc(EffRk)+1]*(EffRk-trunc(EffRk)))^(1/EffRk)
  RelDisp <- (sqrt(EffRkSGV))/(sqrt((1)/(pi)*(EffRk)))
  RelDisp
}

#Simulation
library(MASS)
p <- 30
n <- seq(5,250, by = 15)
nsets <- 1000
ICV.n <- VE.n <- RelVE.n <- T1.n <- T2.n <- RelDisp.n <- array(NA,dim = c(nsets,length(n)))
for (j in 1:length(n)){
  mydat <-lapply(1:nsets, function(i) mvrnorm(n = n[j], mu = rep(0,p),
                                               Sigma = diag(1,p)))
  eigs <-lapply(1:nsets, function(i)  eig.zap(mydat[[i]]))
  ICV.n[,j] <- unlist(lapply(1:nsets, function(i) ICV(eigs[[i]])))
  VE.n[,j] <- unlist(lapply(1:nsets, function(i) VE(eigs[[i]])))
  RelVE.n[,j] <- unlist(lapply(1:nsets, function(i) RelVE(eigs[[i]])))
  T1.n[,j] <- unlist(lapply(1:nsets, function(i) T1(eigs[[i]])))
  T2.n[,j] <- unlist(lapply(1:nsets, function(i) T2(eigs[[i]])))
  RelDisp.n[,j] <- unlist(lapply(1:nsets, function(i) RelDisp(eigs[[i]])))
}

p <- seq(5,250, by = 15)
n <- 200
nsets <- 1000
ICV.p <- VE.p <- RelVE.p <- T1.p <- T2.p <- RelDisp.p <- array(NA,dim = c(nsets,length(p)))
for (j in 1:length(p)){
  mydat <-lapply(1:nsets, function(i) mvrnorm(n = n, mu = rep(0,p[j]),
                                               Sigma = diag(1,p[j])))
  eigs <-lapply(1:nsets, function(i)  eig.zap(mydat[[i]]))
```

```
  ICV.p[,j] <- unlist(lapply(1:nsets, function(i) ICV(eigs[[i]])))
  VE.p[,j] <- unlist(lapply(1:nsets, function(i) VE(eigs[[i]])))
  RelVE.p[,j] <- unlist(lapply(1:nsets, function(i) RelVE(eigs[[i]])))
  T1.p[,j] <- unlist(lapply(1:nsets, function(i) T1(eigs[[i]])))
  T2.p[,j] <- unlist(lapply(1:nsets, function(i) T2(eigs[[i]])))
  RelDisp.p[,j] <- unlist(lapply(1:nsets, function(i) RelDisp(eigs[[i]])))
}

F1.sim.res <- list(ICV.n = ICV.n, VE.n = VE.n, RelVE.n = RelVE.n, T1.n = T1.n,
                   T2.n = T2.n, ICV.p = ICV.p, VE.p = VE.p, RelVE.p = RelVE.p,
                   T1.p=T1.p,T2.p=T2.p, RelDisp.p = RelDisp.p, RelDisp.n = RelDisp.n)
save(F1.sim.res, file = "Fig1.results.Rdata")
```

3.2: Properties of V_{rel} and Z_{Vrel}

In the main article, we evaluated several additional properties of V_{rel}, including its ability to estimate known levels of input integration, and the variance of its sampling distribution. The following r-scripts were used to generate Figures 3 and 4, which contained those results.

```
library(MASS)
source('WatanabeCov.r')
source('Zr-Functions.R')

#Across N (RxC = N x r)
p <- 32
n <- c(8,16,32,64,128,256)
r <- c(.1,.3,.6,.9)
nsets <- 1000
Vrel.N <- Zrel.N <- array(NA,dim=c(length(r),length(n),nsets))
for(i in 1:length(r)){
  Sigma <- GenCov(p, VR = r[i], shape = "q-large")
  for(j in 1:length(n)) {
    dat <- lapply(1:nsets, function(k) {
      mvrnorm(n = n[j], mu = rep(0,p), Sigma = Sigma)
    })
    out <- lapply(1:nsets, function(k) { Z.Vrel(dat[[k]])  })
    Vrel.N[i,j,] <- unlist(lapply(1:nsets, function(k) {out[[k]]$Re.obs}))
    Zrel.N[i,j,] <- unlist(lapply(1:nsets, function(k) {out[[k]]$ZR}))
  }
}
Vrel.mn.N <- apply(Vrel.N,c(1,2),mean)
Vrel.sd.N <- apply(Vrel.N,c(1,2),sd)
ZR.mn.N <- apply(Zrel.N,c(1,2),mean)
ZR.sd.N <- apply(Zrel.N,c(1,2),sd)

#Across p (RxC = p x r)
p <- c(8,16,32,64,128,256)
n <- 128
r <- c(.1,.3,.6,.9)
nsets <- 1000
Vrel.p <- Zrel.p <- array(NA,dim=c(length(r),length(p),nsets))
for(i in 1:length(r)){
  for(j in 1:length(p)) {
```

```
    Sigma <- GenCov(p[j], VR = r[i], shape = "q-large")
    dat <- lapply(1:nsets, function(k) {
      mvrnorm(n = n, mu = rep(0,p[j]), Sigma = Sigma)
    })
    out <- lapply(1:nsets, function(k) { Z.Vrel(dat[[k]])  })
    Vrel.p[i,j,] <- unlist(lapply(1:nsets, function(k) {out[[k]]$Re.obs}))
    Zrel.p[i,j,] <- unlist(lapply(1:nsets, function(k) {out[[k]]$ZR}))
  }
}
Vrel.mn.p <- apply(Vrel.p,c(1,2),mean)
Vrel.sd.p <- apply(Vrel.p,c(1,2),sd)
ZR.mn.p <- apply(Zrel.p,c(1,2),mean)
ZR.sd.p <- apply(Zrel.p,c(1,2),sd)

F2.res <- list(Vrel.mn.N = Vrel.mn.N,  Vrel.sd.N = Vrel.sd.N, ZR.mn.N = ZR.mn.N,
               ZR.sd.N = ZR.sd.N, Vrel.mn.p = Vrel.mn.p,  Vrel.sd.p = Vrel.sd.p,
               ZR.mn.p = ZR.mn.p, ZR.sd.p = ZR.sd.p)

save(F2.res, file = "Fig2-3.AB.results.Rdata")

#2C/3C
p <- 32
n <- 128
r <- c(.1,.3,.5,.7,.9)
nsets <- 2500
Vrel.2C <- Zrel.2C <- NULL
for(i in 1:length(r)){
  Sigma <- GenCov(p, VR = r[i], shape = "q-large")
  dat <- lapply(1:nsets, function(j) {
    mvrnorm(n = n, mu = rep(0,p), Sigma = Sigma) })
  out <- lapply(1:nsets, function(j) { Z.Vrel(dat[[j]])  })
  Vrel.2C <- cbind(Vrel.2C,unlist(lapply(1:nsets, function(j) {out[[j]]$Re.obs})))
  Zrel.2C <- cbind(Zrel.2C,unlist(lapply(1:nsets, function(j) {out[[j]]$ZR})))
}

#2D
p <- 32
n <- 128
r <- seq(0.01,0.99,by =.02)
nsets <- 1000
Vrel.var <- array(NA,dim=length(r))
for(i in 1:length(r)){
  Sigma <- GenCov(p, VR = r[i], shape = "q-large")
  dat <- lapply(1:nsets, function(j) {
    mvrnorm(n = n, mu = rep(0,p), Sigma = Sigma) })
  Vrel.var[i] <- var(unlist(lapply(1:nsets, function(j) { Z.Vrel(dat[[j]])$Re.obs  })))
}

F2CD.res <- list(Vrel.2C = Vrel.2C,  Zrel.2C = Zrel.2C, Vrel.var = Vrel.var)

save(F2CD.res, file = "Fig2.CD.results.Rdata")
```

3.3: Statistical Properties of Z_{Vrel}

In the main article, we evaluated the statistical performance of two-sample tests based on Z_{Vrel}. The following r-scripts were used to generate Figure 5, which contained those results.

```
library(MASS)
source('WatanabeCov.r')
source('Zr-Functions.R')

### Fig 4A: Error Rates
runs <- 100
p <- 16
n <- 128
nset <- 1000
r <- c(0.0,0.0,0.3,0.6,0.6)

Prop.sig <- matrix(NA,runs,4)
for (ii in 1:runs){
  Sigma <- lapply(1:length(r), function(i) {
    Sigma <- GenCov(p, VR = r[i], shape = "q-large")
  })
  dat <- lapply(1:nset, function(i){
    x1 <- lapply(1:length(r), function(j) {mvrnorm(n = n, mu = rep(0,p),
                                                   Sigma = Sigma[[j]])   })
  })
  results <- lapply(1:nset, function(j){
    Z <- lapply(1:length(r), function(i){
      Z.Vrel (dat[[j]][[i]])
    })
    N.N <- compare.ZR(Z[[1]],Z[[2]])$pairwise.P[2,1]
    Y.Y <- compare.ZR(Z[[4]],Z[[5]])$pairwise.P[2,1]
    N.Y <- compare.ZR(Z[[1]],Z[[5]])$pairwise.P[2,1]
    Sm.Y <- compare.ZR(Z[[3]],Z[[5]])$pairwise.P[2,1]
    res <- c(N.N,Y.Y,Sm.Y,N.Y)
    res
  })

  Pvals <- simplify2array(results)
  Prop.sig[ii,] <- rowSums(ifelse(Pvals<=0.05, 1,0)) / nset
  cat(paste("run#", ii,sep = ""))
}
save(Prop.sig, file="Error.results.Rdata")

### Fig 4B: Power Curves
p <- 16
n <- c(16,32,64,128,256)
nset <- 1000
r <- c(0.0,0.0,0.2,0.4,0.6,0.8, 0.99)

Prop.sig <- matrix(NA,length(n),6)
for (ii in 1:length(n)){
  Sigma <- lapply(1:length(r), function(i) {
    Sigma <- GenCov(p, VR = r[i], shape = "q-large")
  })
```

```
  dat <- lapply(1:nset, function(i){
    x1 <- lapply(1:length(r), function(j) {mvrnorm(n = n[ii], mu = rep(0,p),
                                                     Sigma = Sigma[[j]])   })
  })
  results <- lapply(1:nset, function(j){
    Z <- lapply(1:length(r), function(i){
      Z.Vrel(dat[[j]][[i]])
    })
    R.0 <- compare.ZR(Z[[1]],Z[[2]])$pairwise.P[2,1]
    R.2 <- compare.ZR(Z[[1]],Z[[3]])$pairwise.P[2,1]
    R.4 <- compare.ZR(Z[[1]],Z[[4]])$pairwise.P[2,1]
    R.6 <- compare.ZR(Z[[1]],Z[[5]])$pairwise.P[2,1]
    R.8 <- compare.ZR(Z[[1]],Z[[6]])$pairwise.P[2,1]
    R.99 <- compare.ZR(Z[[1]],Z[[7]])$pairwise.P[2,1]
    res <- c(R.0,R.2,R.4,R.6,R.8,R.99)
    res
  })
  Pvals <- simplify2array(results)
  Prop.sig[ii,] <- rowSums(ifelse(Pvals<=0.05, 1,0)) / nset
  cat(paste("run#", ii,sep = ""))
}
save(Prop.sig, file="PowerCurves.P16.Rdata")
```

References

O'Keefe, F. R., J. A. Meachen, and P. D. Polly. 2022. On information rank deficiency in phenotypic covariance matrices. Systematic Biology 71:(In Press).

Watanabe, J. 2022. Statistics of eigenvalue dispersion indices: Quantifying the magnitude of phenotypic integration. Evolution 76:4–28.
